# Migration of Deep Learning Models Across Ultrasound Scanners

**DOI:** 10.1109/TBME.2025.3564567

**Published:** 2025-11

**Authors:** Ufuk Soylu, Varun Chandrasekeran, Gregory J. Czarnota, Michael L. Oelze

**Affiliations:** Department of Electrical and Computer Engineering, University of Illinois at Urbana-Champaign, USA; Department of Electrical and Computer Engineering, University of Illinois at Urbana-Champaign, USA; Sunnybrook Health Sciences Centre, Canada; Department of Electrical and Computer Engineering, University of Illinois at Urbana-Champaign, Urbana, IL 61801 USA, and also with the Beckman Institute for Advanced Science and Technology and the Carle Illinois College of Medicine, University of Illinois at Urbana-Champaign, Urbana, IL 61801 USA

**Keywords:** Tissue characterization, deep learning, ultrasound imaging, deep model security, transfer function, data mismatch

## Abstract

A transfer function approach has recently proven effective for calibrating deep learning (DL) algorithms in quantitative ultrasound (QUS), addressing data shifts at both the acquisition and machine levels. Expanding on this approach, we develop a strategy to acquire the functionality of a DL model from one ultrasound machine and implement it on another in a black-box setting, in the context of QUS. This demonstrates the ease with which the functionality of a DL model can be transferred between machines. While the proposed approach can also assist regulatory bodies in comparing and approving DL models, it also highlights the security risks associated with deploying such models in a commercial scanner for clinical use. The method is a black-box unsupervised domain adaptation technique that integrates the transfer function approach with an iterative schema. It does not utilize any information related to model internals but it solely relies on the availability of an input-output interface. Additionally, we assume the availability of unlabeled data from a testing machine. This scenario could become relevant as companies begin deploying their DL functionalities for clinical use. In the experiments, we used a SonixOne and a Verasonics machine. The model was trained on SonixOne data, and its functionality was then transferred to the Verasonics machine. The proposed method successfully transferred the functionality to the Verasonics machine, achieving a remarkable 98 percent classification accuracy in a binary decision task. This study underscores the need to establish security measures prior to deploying DL models in clinical settings.

## I. INTRODUCTION

THE integration of deep learning (DL) based biomedical ultrasound has the potential to enhance the quality of medical services through automation and efficiency. Therefore, the integration of DL-based biomedical ultrasound imaging into clinical practice has become the coveted goal for both industry and academia [1], [2], [3], [4], [5]. Thanks to substantial efforts dedicated to overcoming challenges posed by DL-based models in clinical settings, this goal is now within reach. The primary technical challenges stem from data scarcity, attributed to the high costs involved in conducting laboratory experiments, acquiring human image data, and acquiring expert annotations of data. Further exacerbating this is the presence of data mismatch, arising from inevitable discrepancies between the development and clinical environments [6]. These challenges have been well studied and several methods have been proposed to overcome these difficulties [7], [8], [9], [10], [11], [12], [13], [14], [15]. Although ongoing efforts persist in addressing these challenges, DL-powered algorithms have reached a stage of maturity, and increased adoption of such algorithms will occur clinically.

The field of quantitative ultrasound (QUS) has also shifted from traditional methods to DL-based approaches, following the general trend. Specifically, in several recent examples, DL models have demonstrated superior performance to traditional QUS approaches in classifying tissue states [16], [17], [18], [19], [20], [21], [22]. Accordingly, substantial efforts dedicated to overcoming challenges related to clinical adoption, namely data scarcity and data mismatches, have been investigated [7], [8], [9], [10], [11], [12], [13], [23]. Observing the pace of the development in this field, it is reasonable to claim that DL-based QUS approaches in clinical settings are becoming a reality rather than a distant goal.

Given the advancement, there could still be concerns or undiscovered challenges on the path that can delay the wide adoption of DL-based QUS methods in the clinic. In this work, we highlight one such concern: the ease with which the functionality of DL-based methods can be stolen from one machine and implemented on another competitor machine. Previous research by Huang et al. [24] demonstrated that post-processing techniques used to enhance image quality in ultrasound scanners can be stolen under black-box constraints. The current work focuses on DL-based QUS methods and their vulnerability to functionality theft under black-box constraints. There can be great expense associated with the development of DL models for medical diagnostics. Specifically, a major expense is in the accumulation of large amounts of data with expert annotation and labeling. The cost of labeling copious amounts of data for DL training can be expensive and take years of investment. Thomas et al. [25] developed an economical model that predicts 60 to 90 percent of development costs are purely data costs for an average computer vision task. In the medical field, one could estimate that this would go up due to more limitations on data acquisition and expert annotation. Companies investing in the development of DL-based functionalities may be deterred from deploying their DL-based models to their own machine and making it available for clinical use if competitors can effortlessly access their machine and transfer the functionality of these models. Therefore, as an outcome of this work, we highlight the necessity of improving DL model security in the context of biomedical ultrasound imaging by demonstrating the ability to purloin a DL model from one machine, i.e., the victim machine, and deploy the DL model on a different machine, i.e., the perpetrator machine.

The most relevant framework to this study is black-box unsupervised domain adaptation (UDA). In black-box UDA, we do not have access to the model’s internals which means that its weights, architecture, training process, and training data are unknown. However, we have access to the input-output interface, and there is unlabeled data available for the machine to which we aim to transfer functionality. This is the most relevant framework for security threats in deployment of DL-based models in the clinic. From the perspective of competitors, the process of acquiring the model, which can be called “stealing”, can be accomplished through a simple stepwise process. First, the competitor acquires the victim machine, gaining access to its input-output interface. Next, the competitor acquires unlabeled data utilizing their own machine in the pursuit of their own model development, making unlabeled data abundant. The unlabeled data or images from the perpetrator machine are transferred to the victim machine, i.e., test-time calibration [23]. Specifically, image data generated from the perpetrator machine could be transferred to the hard drive of the victim machine assuming the file structure can be replicated so that the victim machine reads the image data. Labels are given to each image from the DL model on the victim machine. These labels are then used in the perpetrator machine to train the model.

To be more precise, in this work, it was assumed that the perpetrator has access to the input-output interface of the victim machine and knows its data format. The focus was on developing a systematic way to transfer the DL capabilities of the victim to the perpetrator. In the scenario where the developed approach is most applicable, there are two competing companies aiming to implement DL capabilities in their machines. Each company possesses the resources and engineering capabilities needed to develop their DL capabilities, including software engineering skills such as data format conversion and configuring input-output interfaces, even in the case of a closed victim system. Overall, this paper highlights a shortcut to acquire the leading company’s DL capabilities and integrate them into the perpetrator’s machine when the leading company makes their DL capabilities clinically available.

The proposed approach could potentially be used by regulatory bodies to compare and approve DL models without needing access to the model’s internals or training data. As DL models become commonplace, regulatory bodies will need effective techniques to evaluate DL models. They can develop regulatory models based on proprietary models. In terms of the “stealing” scenario, the regulatory models correspond to the perpetrator models and the proprietary models correspond to the victim models. In this paper, the terms “victim” and “perpetrator” are utilized rather than “regulatory” and “proprietary”; however, these terminologies can be interchangeable depending on the scenario in mind. Regulatory bodies can then use regulatory models, which have open weights, for their investigation, comparison, and approval process.

A comprehensive literature survey on adaptation algorithms at test time can be found in [26]. Black-box UDA falls under the umbrella of test-time adaptation. The main idea in test-time adaptation is to adapt a given pre-trained model, which was trained in a supervised manner on the training domain, to the test domain without requiring labeled data. There exist multiple strategies to address the black-box UDA problem: pseudo-labeling aims to assign labels for unlabeled data via the black-box model. Consistency regularization aims to enforce consistent network predictions by adding a loss term in the training. Clustering assumes that the decision boundary in the unlabeled data should lie in low-density regions. Self-supervised learning aims to learn feature representation from unlabeled data based on an auxiliary prediction task that is then used in the downstream task. Given the vast literature, iterative learning with noisy labels (IterLNL) [27] is a state-of-the-art black-box UDA method which conducts noisy labeling and learning with noisy labels, iteratively. Noisy labels are referred to as noisy because the labeling processes in the test domain make inevitable mistakes due to domain mismatch when utilizing the pre-trained model from the training domain. In this work, we demonstrate that combining IterLNL with our transfer function approach [7], [23] leads to successful transfer of the DL functionality from a victim machine to a perpetrator machine. The transfer function approach was developed and proven to mitigate domain mismatch issues in ultrasound imaging systems due to hardware or imaging parameter mismatches in the context of QUS. In combination with IterLNL, the classification accuracy for the perpetrator machine reached 98 percent.

**Fig. 1.**
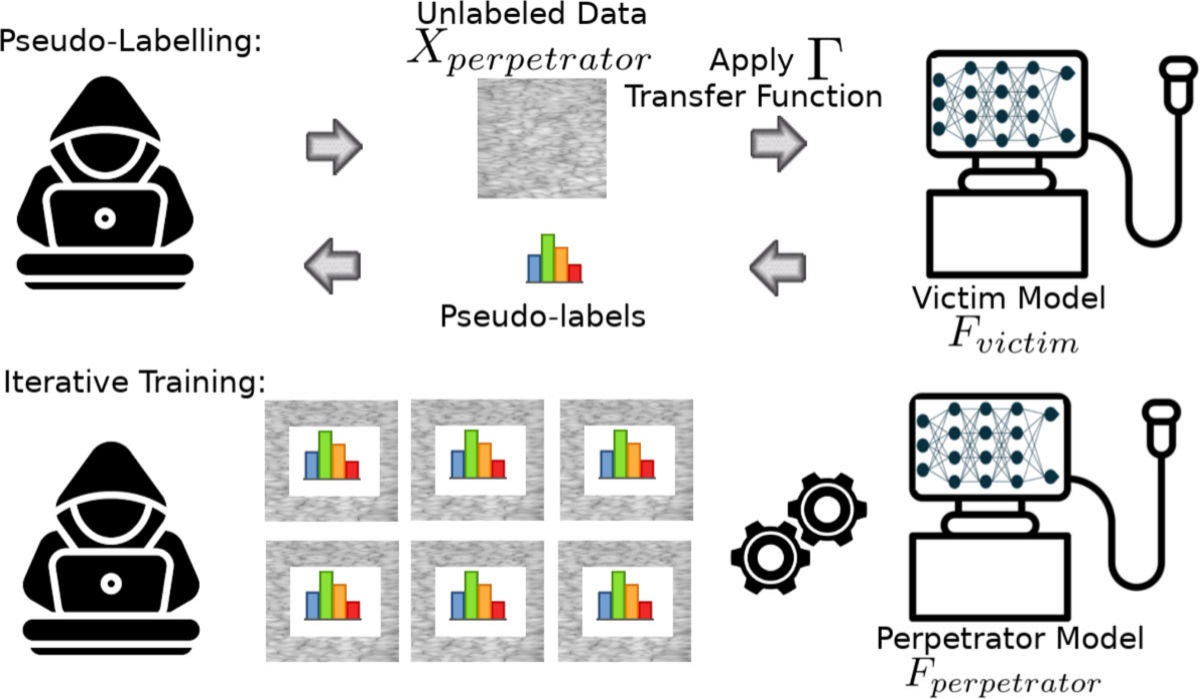
Overall method in two stages: Pseudo-labeling, the perpetrator obtains pseudo-labels from the victim machine for the unlabeled data, utilizing a transfer function. Subsequently, Training, the perpetrator iteratively trains their model using the noisy labels obtained in the pseudo-labeling stage.

The proposed method, depicted in Fig. 1, utilizes a transfer function at test time to calibrate mismatches between the victim and the perpetrator machines. Subsequently, pseudo-labels can be obtained for unlabeled data from the perpetrator machine through the input-output interface of the victim machine. These labels can still be noisy. Therefore, we propose utilizing IterLNL to further refine the labels. Then, a new DL model can be trained for the perpetrator machine utilizing these refined labels, which copies the functionality of the DL-based model from the victim to the perpetrator machine. The proposed method accesses only the input-output interface without accessing model internals of the victim machine, which are presumed not to be available. Another note is that the transfer function method requires the acquisition of calibration data using the victim and the perpetrator machines with a calibration phantom. Further details of the proposed approach will be discussed in Section II-A. In Sections II and III, our methodology and experimental results are detailed, respectively. Following this, we provide discussion and conclusions in Sections IV and V, respectively.

## II. METHODS

### A. Proposed Approach

The proposed approach combines the transfer function approach [7] with IterLNL [27]. The transfer function is able to calibrate data mismatches between ultrasound machines over a defined bandwidth. The transfer function has been shown to mitigate acquisition-related and hardware-related mismatches in a systematic way between two systems. It has been demonstrated that utilizing the transfer function between two machines at test time, i.e., converting data acquired on one machine to the data that could be used for a DL model developed on another machine, achieved approximately an AUC score of 0.99 and an accuracy score of 80 percent for the QUS task of interest in this work [23]. Therefore, as a refinement step, IterLNL, which conducts iterative learning with noisy labels, is performed to further refine the labels. Following this, a new learning algorithm can be developed using these refined labels for the perpetrator machine. Therefore, the proposed approach at a high level involves five steps:

1. Gather calibration data using the victim machine and the perpetrator machine.
2. Calculate the transfer function between the perpetrator and victim machines.
3. Obtain pseudo-labels for the unlabeled data from the perpetrator machine utilizing the transfer function and the input-output interface of the victim machine.
4. Implement iterative learning with pseudo-labels for refinement.
5. Obtain the learning algorithm for the perpetrator machine using the refined pseudo-labels.

The transfer function approach at test time exploits the decomposition of an ultrasound image’s frequency spectrum into the tissue signal and the system response,

(1) $$I_{ultrasound}(\text{f},\text{x})=S_{\phi_{machine}}(\text{f},\text{x})P_{tissue}(\text{f},\text{x})$$

where $I_{ultrasound}$ is the ultrasound’s image frequency spectrum, x is the axial location, f is the transmit frequency $S_{\phi_{machine}}$ is the system response, and $P_{tissue}$ is the imaging substrate. Then, the transfer function can be calculated using a calibration phantom and obtaining a single view from a calibration phantom utilizing both machines,

(2) $$\frac{I_{victim\;}(\text{f},\text{x})}{I_{perpetrator\;}(\text{f},\text{x})}=\frac{S_{\phi_{victim\;}}(\text{f},\text{x})}{S_{\phi_{perpetrator\;}}(\text{f},\text{x})}$$

(3) =Γ

where Γ is the transfer function at test time. Following the methodology of the original transfer function work, we computed the transfer function at various depths and employed the Wiener implementation of it,

(4) $$\Gamma^{Wiener\;}=\frac{|\Gamma {|}^{-1}}{|\Gamma {|}^{-2}+SNR^{-1}}.$$

For simplicity, $\Gamma^{Wiener\;}$ will be referred as Γ for the rest of the paper. SNR was also computed at various depths, utilizing the power spectra of $I_{victim\;}$ and $I_{perpetrator\;}$ in the calibration data, by determining a noise floor level outside of the analysis bandwidth for the transducers used in the experiments. Actually, we obtained the transfer function in two ways: stable acquisition, which involved capturing 10 calibration views from a fixed location of the calibration phantom, and free-hand acquisition, which involved recording a video of calibration frames using free-hand motion. For the stable acquisition, by securing the transducer using a bar clamp holder, identical calibration frames were captured from precisely the same position on the calibration phantom. Multiple frames were recorded to mitigate or reduce any potential effects of electrical noise through averaging. On the other hand, for the free-hand acquisition, a video of 1000 frames was recorded. While stable acquisition is the most standard way to obtain calibration data, free-hand acquisition is necessary in scenarios where stable acquisition is not possible. In this study, in one of the experiments, we utilized different transducers for the victim and perpetrator machines and had to implement free-hand acquisition for the calibration data.

The key idea in IterLNL is to iteratively learn a new model using noisy labels, based on anchors, which are data points with the lowest training loss. Let’s denote the victim model as $F_{victim\;}$, the unlabeled data as $X_{perpetrator\;}$, the perpetrator model as $F_{perpetrator\;}$, and the anchors as $U_{perpetrator\;}$.

Pseudo-labels $y_{perpetrator\;}$ are obtained by applying the input-output interface of $F_{victim\;}$ to $\Gamma \left(X_{perpetrator\;}\right)=X_{perpetrator\;\to \;victim\;\;}$. During each denoising cycle, pseudo-labels are re-acquired using the learned perpetrator model $F_{perpetrator\;}$. An important parameter in IterLNL is the noise rate, denoted as ϵ. In the original work, it was estimated using $y_{perpetrator\;}$ and $X_{perpetrator\;\to \;victim\;\;}$. However, in this study, we used prior knowledge of the transfer function to set the noise rate at approximately 20 percent. The noise rate estimates the noise in $y_{perpetrator\;}$ and determines the anchors. In this study, anchors are 80 percent of the data points with the lowest training loss, representing correct labels.

**Algorithm 1:** Proposed Approach.

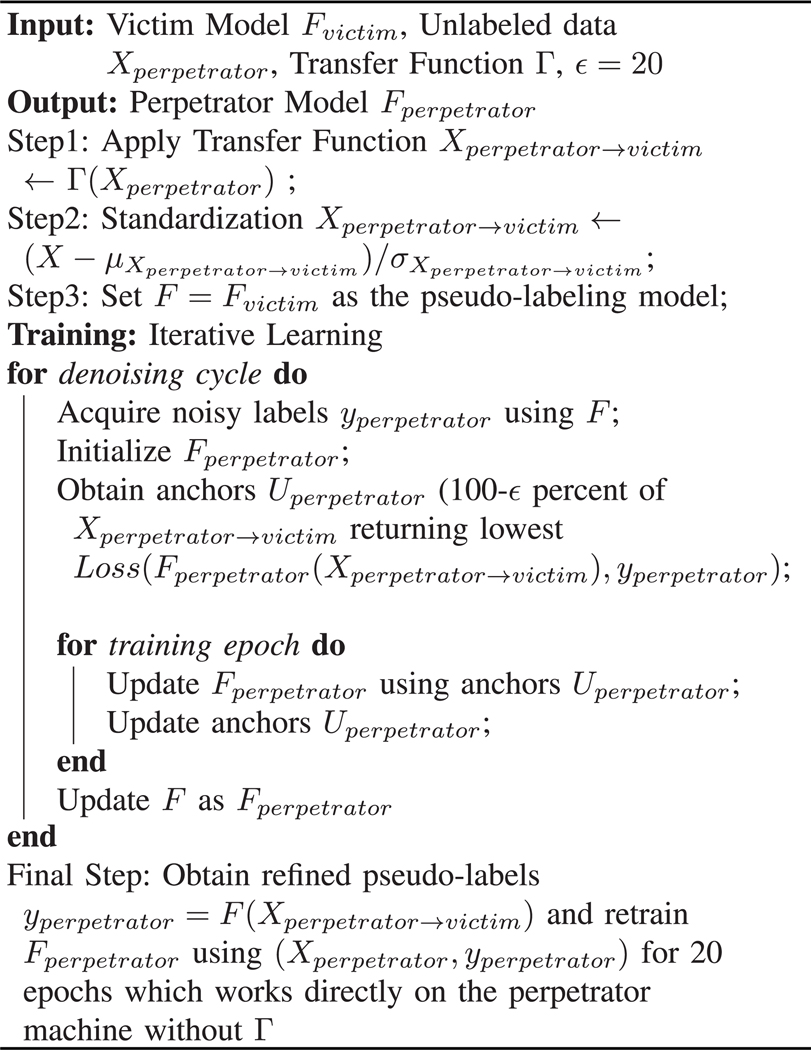

The proposed approach, combining the transfer function and IterLNL, is depicted in Algorithm 1. After the iterative learning process, $F_{perpetrator\;}$ can generate highly accurate labels for the transformed data $X_{perpetrator\;\to \;victim\;\;}$. Finally, $F_{perpetrator\;}$ is updated to operate directly within the perpetrator machine domain by training it on $X_{perpetrator\;}$ without the transfer function Γ.

### B. Priors

The proposed method utilized two information priors: label distribution and noise rate ϵ. We utilized the prior for the label distribution to obtain noisy labels through the pseudo-labeling model. In our experimental setup, the DL models generated a single score. When the class score approached 0, the likelihood of it being class 0 increased. When the score approached 1, the likelihood of it being class 1 increased. Following that, in Algorithm 1, during the acquisition of noisy labels $y_{perpetrator\;}$ using F, we employed our prior knowledge of the label distribution to set the threshold between class 0 and 1. As we operated within a balanced binary classification setup, we determined the threshold as the median value (50^*th*^ percentile). On the other hand, we utilized the prior of noise rate in determining anchors to be used in training. Based on previous findings [23], the average accuracy for the transfer function at test time was approximately 80 percent, indicating a noise rate of 20 percent. Note that this noise rate is actually conservative, as the 80 percent accuracy was obtained without any label prior in the training or evaluation. In Section III, we experimented with these priors to simulate scenarios when there is approximate or vague information on these priors.

### C. Phantoms

**Fig. 2.**
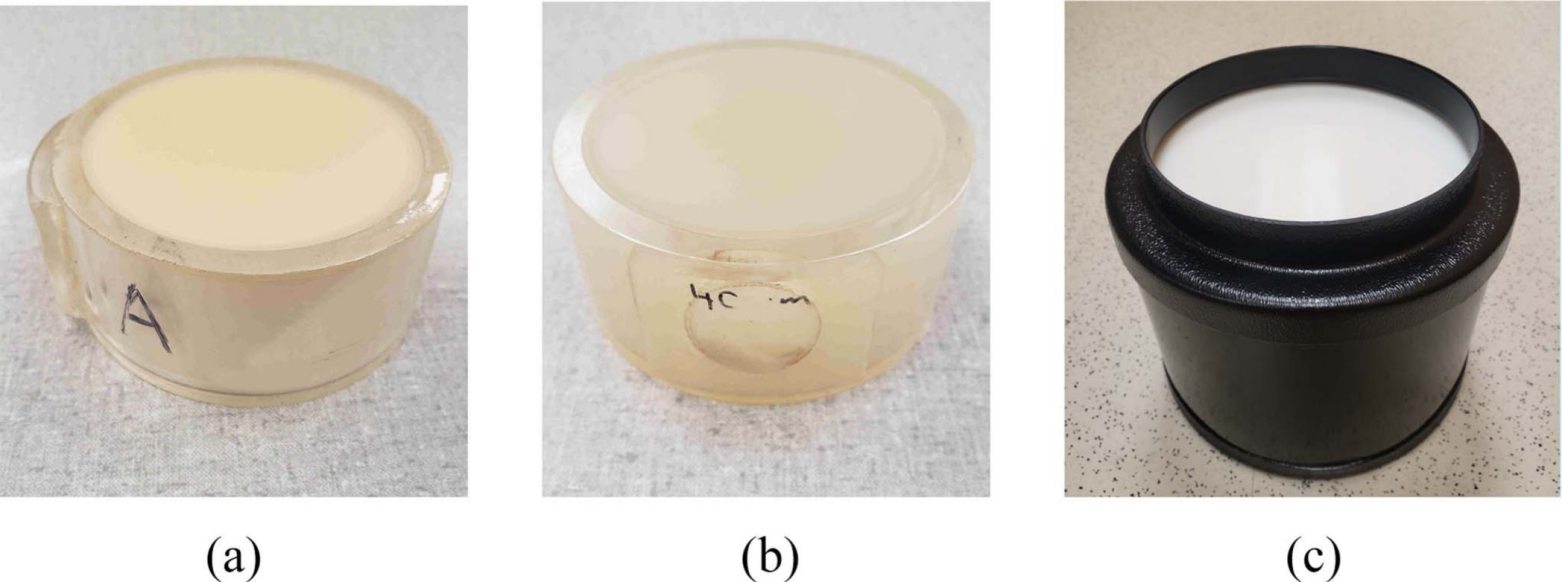
Phantoms in the experiments: (a) Classification Phantom 1, (b) Classification Phantom 2, and (c) The Calibration Phantom.

The experiments used one calibration phantom and two classification phantoms with their photographs shown in Fig. 2. The QUS task in this study was to determine the class identity of the ultrasound data from each phantom. Clinically, this could correspond to a binary tissue classification problem, e.g., is the tissue diseased or not diseased, or is the the tumor malignant or benign, or is the liver fatty or normal. However, in this study we limit the application to classifying between two phantoms to validate the approach. The objective of the study is to acquire this functionality from the victim machine and implement it on the perpetrator machine.

A commercial QUS reference phantom from CIRS, Inc., Norfolk, VA (part no. 14090502, serial no. 221447541) was used as the calibration phantom. The phantom had a speed of sound (SOS) of 1545 meters per second and an attenuation coefficient slope (ACS) of 0.74 dB per centimeter per megahertz.

Classification Phantom 1 was designed to mimic the characteristics of the human liver [28], with construction details provided in [29]. It contained glass-bead scatterers with diameters ranging from 75 to 90 *μ* m in diameters. The phantom had an ACS of 0.4 dB per centimeter per megahertz, with a SOS of 1540 meters per second.

Classification Phantom 2 was designed to have low attenuation characteristics [30], with construction details provided in [31]. It contained glass-bead scatterers with diameters ranging from 39 to 43 *μ* m. The phantom had an ACS of 0.1 dB per centimeter per megahertz, with a SOS of 1539 meters per second.

### D. Ultrasound Machines

Two ultrasound machines, a SonixOne system (BK Ultrasound, Boston, MA, USA) and a Verasonics Vantage 128 system (Verasonics, Kirkland, WA, USA), were utilized. These machines provide a research interface that allows the extraction of RF data.

The victim model was trained using data from the SonixOne system, which designated it as the victim machine. The perpetrator model aimed to replicate the same functionality as the victim model but for the Verasonics system, positioning it as the perpetrator machine. Throughout the experiments, an L9–4 transducer and an L11–5 transducer were utilized. The L9–4 transducer was used for the SonixOne system, while both the L9–4andL11–5transducerswereusedfortheVerasonicssystem.

To prepare the perpetrator image data from the Verasonics system for the input-output interface on the victim machine, it was necessary to adjust the sampling rate mismatch between the two machines. Although the Verasonics machine offers various sampling rates, it did not offer the same rate as the SonixOne. Therefore, the Verasonics machine captured raw channel data at a 50 MHz sampling rate, while the SonixOne machine obtained radio-frequency (RF) data at a 40 MHz sampling rate after it had been beamformed. These differences were mitigated by preprocessing the Verasonics data. First, delay-and-sum beamforming was applied to the raw channel data. Next, a multirate finite impulse response filter with an upsampling factor of 4 and a downsampling factor of 5 was used to correct the sampling rate mismatch. All these preprocessing operations were done on Matlab, specifically using the ‘designMultirateFIR’ and the ‘dsp.FIRRateConverter’ functions. This way, the Verasonics data was matched to the SonixOne data in terms of sampling rate. The victim model was trained using 1000 frames per classification phantom, totaling 2000 frames, acquired using the SonixOne machine. Similarly, for the perpetrator model, 2000frameswereacquiredusingtheVerasonicsmachine.These acquisitions involved recording a video of frames while moving the transducer across the phantom surface.

Linear sequential scanning was conducted for both machines. During acquisition, a single axial focus was set at 2 cm. The Verasonics system operated at a pulse frequency of 5 MHz and an transmit voltage of 45.2 Volts. In contrast, the SonixOne system operated at a pulse frequency of 9 MHz and an output power of 100 percent. Note that, between the perpetrator and the victim machines, there are acquisition-related mismatches such as transmit power and transmit pulse frequency, in addition to hardware-related mismatches. The use of the transfer function in the proposed approach, when obtaining $X_{perpetrator\;\to \;victim\;}$, is essential to mitigate these mismatch types. As the calibration data from the victim machine was collected at a pulse frequency of 9 MHz and an output power of 100 percent, and from the perpetrator machine at a pulse frequency of 5 MHz and a transmit voltage of 45.2 volts, the resulting transfer function was capable of mitigating the acquisition mismatches.

### E. Data Preparation

An ultrasound image frame from both machines was 2080 pixels in height and 256 pixels in width, corresponding to 4 cm axial depth and 38 mm lateral span with 128 array channels. The victim and the perpetrator model used the RF ultrasound data. From the ultrasound images, rectangular patches were obtained for training and evaluating the DL models. They had a height of 200 samples and a width of 26 samples, equivalent to 4 mm by 4 mm. In traditional QUS, a region of interest in the ultrasound frame is extracted and analyzed. The patch-wise data processing in DL models in this work was inspired by the nature of traditional QUS.

**Fig. 3.**
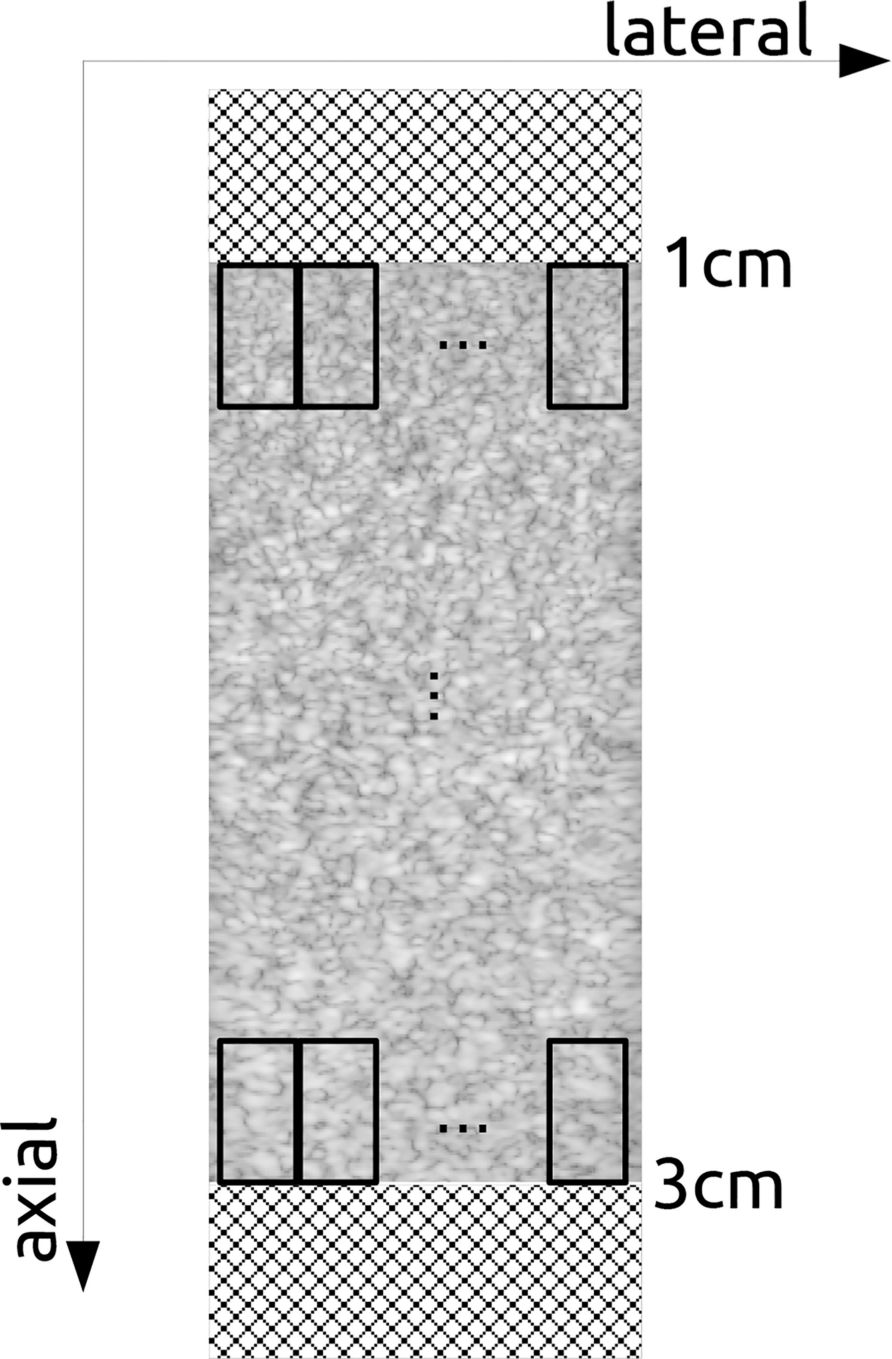
QUS Data Patch Extraction: 81 data patches extracted per frame from 9 axial and 9 lateral lines.

The patch extraction is depicted in Fig. 3. The initial 540 axial pixels were skipped. Axially, the next sequence started after shifting 100 pixels. Laterally, the next sequence started after shifting 26 pixels. This configuration was chosen to extract the same number of patches in both the axial and lateral directions. Overall, 81 patches perultrasound frames were extracted coming from 9 lateral and 9 axial locations.

The 2000 frames from the SonixOne machine were divided into a training set (80 percent) and a validation set (20 percent) for the development of the victim model. On the other hand, for the development of the perpetrator model, 2000 frames from the Verasonics machine were split into a training (50 percent) and a testing set (50 percent). Therefore, in the experiments, the perpetrator model was trained using 1000 frames, with 100 frames as validation data and 900 frames as training data, and it was evaluated using the test set consisting of 1000 ultrasound frames.

### F. Network Structure

The victim model was a DenseNet-201 [32], depicted in Table I, with minor modifications to their input-output relationships tailored to our specific problem. The perpetrator model was chosen as ResNet-34 [33], depicted in Table II, with similar modifications. These modifications involve adjusting the first convolutional layer to accommodate single feature channel inputs and modifying the final output layer, including a sigmoid activation, to output a single score for a binary classification setup. During the initialization of network parameters, pretrained weights from [33] were utilized via PyTorch, with the exception of the first 2D convolution layer and the last linear layer. They were randomly initialized utilizing PyTorch’s default method, which is a uniform distribution bounded by $1/\sqrt{fanin\;}$ for the linear layer, where fanin refers to the number of input features (neurons) before the linear layer, and a uniform distribution bounded by $1/\sqrt{\;fanin\;\;\text{×}\;kernelsize\;}$ for the convolution layer, where kernelsize refers to the dimensions of the convolution filter used in the convolution operation, and fanin refers to the number of input channels multiplied by the area of the convolutional filter. Then, all parameters were unfrozen and updated using backpropagation during training. The necessity of selecting different model architectures between the victim and the perpetrator arose from the black-box nature of the victim setup, resulting in inevitable differences in architectural design. As long as the selected architecture has enough complexity to solve the task, the attack should be successful. As a side note, the criteria for a successful attack could vary depending on the task. In this work, for the classification task at hand, the classification accuracy on the perpetrator machine must be satisfactorily high, for example, 95 percent. In practice, network selection may require search and hyperparameter tuning, especially in a black-box setup. In the scope of thepaper, we assumed two companies with engineering and research capabilities and resources. Given the scenario, the two companies would be able to approximately estimate their competitors’ modeling choices, such as architecture and complexity, at a high level. In the paper, we used two different architectures to reflect the situation that they cannot estimate exactly but approximately.

### G. Training

During training, the Adam algorithm [34] was used as the optimizer. The cross-entropy loss

(5) $$-\left(y_{perpetrator\;}\log(\hat{y})+\left(1-y_{perpetrator\;}\right)\log(1-\hat{y})\right)$$

where $\hat{y}=F_{perpetrator\;}\left(X_{perpetrator\;\to \;victim\;}\right)$, was used for training. Z-score standardization was conducted patch-wise, which involved subtracting the patch-wise mean and normalizing it with the patch-wise standard deviation. A batch size of 2048 patches was employed for all experiments to maximize memory utilization. Data augmentation involved horizontally flipping the patches with a 0.5 probability.

In iterative learning, during each denoising cycle, 10 percent of the data, not necessarily the same portion, was held out for use as a validation partition. This allocation allowed for the observation of training loss and the determination of hyperparameters, such as setting the training epoch number to 10 and the learning rate to 1 × 10^−5^. It was observed that for a given denoising cycle of IterLNL, the training losses converged with the selected hyper-parameter configuration. In the results, the experiments were repeated 10 times starting from the iterative learning phase. Using different random seeds, the entire iteration process was repeated—10 random seeds were used, and for each random seed, we repeated 10 denoising cycles in the iterative learning. This means we had a distribution of results at each iteration with a sample size of 10 due to the 10 random seeds. Subsequently, the performance of the final $F_{perpetrator\;}$ model on the hidden data from the Verasonics machine was evaluated for each random seed, leading to a distribution of evaluation metrics, with a sample size of 10, such as classification accuracy and AUC scores. When reporting the performance metrics, averages and standard deviations were calculated. The performance evaluation metrics included average classification accuracy and AUC score, along with their respective standard deviations. When calculating classification accuracy and AUC score, it was done patch-wise. The observed standard deviation was due to random parameter initialization in each denoising cycle.

For computational resources, four NVIDIA RTX A4000 GPUs in a workstation were employed for data parallelism. The PyTorch library [35] was utilized for the implementation.

## III. RESULTS

The victim model scored an accuracy of 99.93 percent and an AUC of 0.999 on its validation set. This performance serves as the upper bound achievable with the proposed method, acknowledging an anticipated performance degradation due to machine-level mismatches between the victim and perpetrator machines.

Our results can be categorized into four categories. First, we explored the impact of the transfer function in our proposed approach. To examine this, we compared two cases: utilizing the transfer function and omitting it, essentially skipping step 1 in Algorithm 1. Second, our exploration delved into the importance of the priors utilized in the proposed method. These priors covered both the label distribution and the noise rate parameter ϵ. Third, we explored the number of ultrasound frames from the perpetrator machine needed for a successful attack (classification accuracy on the perpetrator machine reached 95 percent). Fourth, we explored the effect of different transducers between the victim and the perpetrator machines.

### A. Ablation Study for the Transfer Function

We conducted an ablation study for the proposed method consisting of two experiments. The first experiment involved using the transfer function within the iterative learning schema. In the second experiment, we omitted the transfer function and solely implemented the iterative learning schema. These experiments were designed to gauge the contribution of the transfer function method. The results for different denoising cycle numbers (2, 5, and 10) are presented in Table III.

### B. Ablation Study for the IterLNL

We conducted an ablation study for the IterLNL in addition to Table III. This study involved a single experiment where the IterLNL was removed from the proposed method, effectively setting the denoising cycle to 0. To ensure a fair comparison, we utilized our information regarding the binary classification setup, i.e. label distribution prior, during the evaluation of this experiment. This means that when obtaining the class labels in the evaluation, half of the lowest scores were assigned to class 0, and the remaining higher scores were assigned to class 1. Then, the accuracy was 95.27 ± 0.07. On the other hand, without using the label distribution prior, in the previous work [23], the accuracy was 80.76 ± 6.87. Together with Table III, these results gauge the contribution of the transfer function and IterLNL separately, in the proposed approach.

### C. Robustness Experiments

We conducted a robustness study for the proposed method consisting of two sets of experiments. In these experiments, we investigated scenarios that involved inaccurate or approximate prior knowledge of the label distribution and noise rate. Specifically, in Table IV, we set the threshold at 40^*th*^ or 60^*th*^ percentile, deviating from 50^*th*^ percentile during the acquisition of noisy labels. To clarify further, this experiment only focuses on changing the threshold for acquiring noisy labels $y_{perpetrator\;}$ using F during iterative learning, and does not change the evaluation. In the evaluation, if the network outputs less than 0.5, it is assigned as class 0, and higher than 0.5 is assigned as class 1. Similarly, in Table V, we examined scenarios where the noise rate was 10 percent or 30 percent, deviating from the established level of 20 percent.

### D. Dataset Size for the Unlabeled Data

**Fig. 4.**
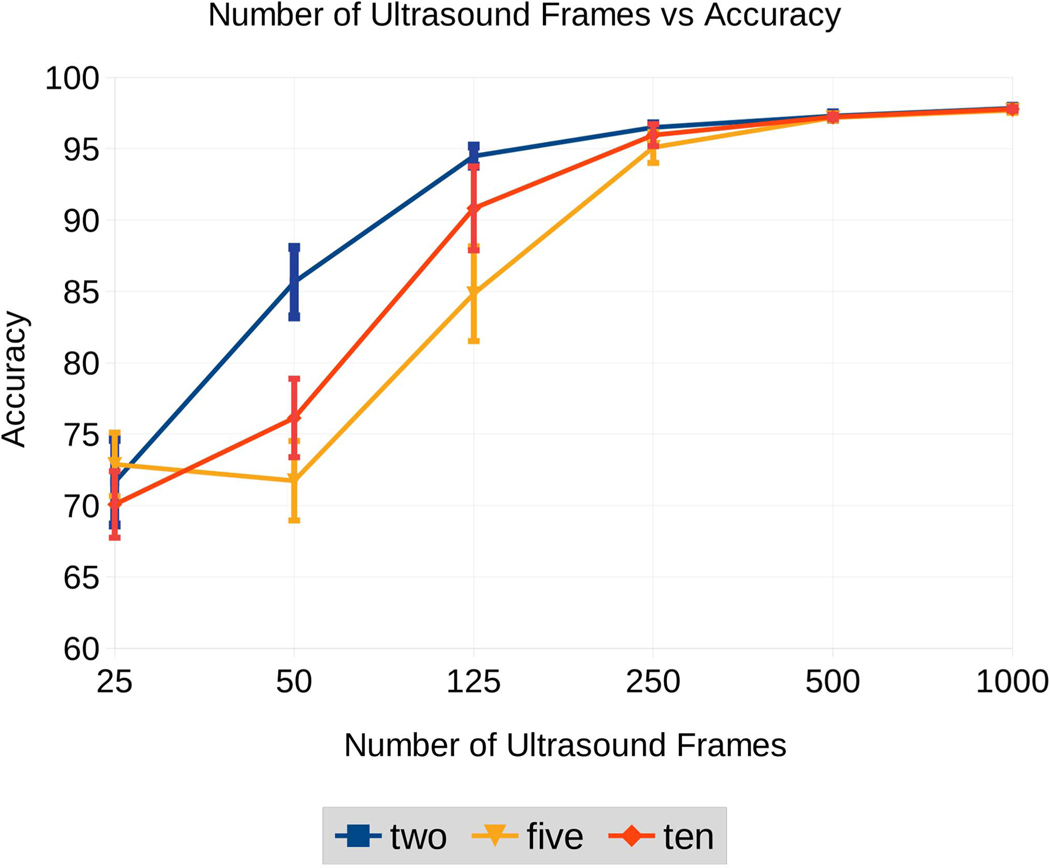
Number of Ultrasound Frames Needed for Successful Attack. Error bars represent one standard deviation. Colors represent different numbers of denoising cycles.

We examined the number of ultrasound frames needed from the perpetrator machines to achieve a successful attack on the victim model. In Fig. 4, on the y-axis, we depicted the classification accuracy achieved on the perpetrator machine using the proposed approach, and on the x-axis, we depicted the number of ultrasound frames utilized from the perpetrator machine. In this result, we performed two, five and ten denoising cycles to refine pseudolabels, setting ϵ to 0.8.

### E. Transducers Mismatch

We examined classification accuracy and AUC when a different transducer was used in the perpetrator machine. This experiment was designed to simulate hardware differences between the victim and the perpetrator machines, and their effect on the proposed approach. In the scope of this paper, there are two competing companies and two different imaging systems. This leads to the question of whether the approach would still be valid if these two machines utilized different transducers. This experiment can be generalized to the broader question of whether these two systems have different hardware components, such as analog-to-digital converters or different processors. We showed that the proposed approach remains effective and raises valid concerns regarding the security aspect.

While the first three sets of experiments solely utilized the L9–4 transducer, this new set of experiments employed the L9–4 transducer for the SonixOne system (perpetrator machine) and the L11–5 transducer for the Verasonics system (victim machine). Note that the imaging configuration was kept the same for the Verasonics system when switching transducers. Table VI reports the results for different denoising cycle numbers. In this experiment, we set ϵ to 0.8.

### F. Example B-Mode Images Before and After the Transfer Function

**Fig. 5.**
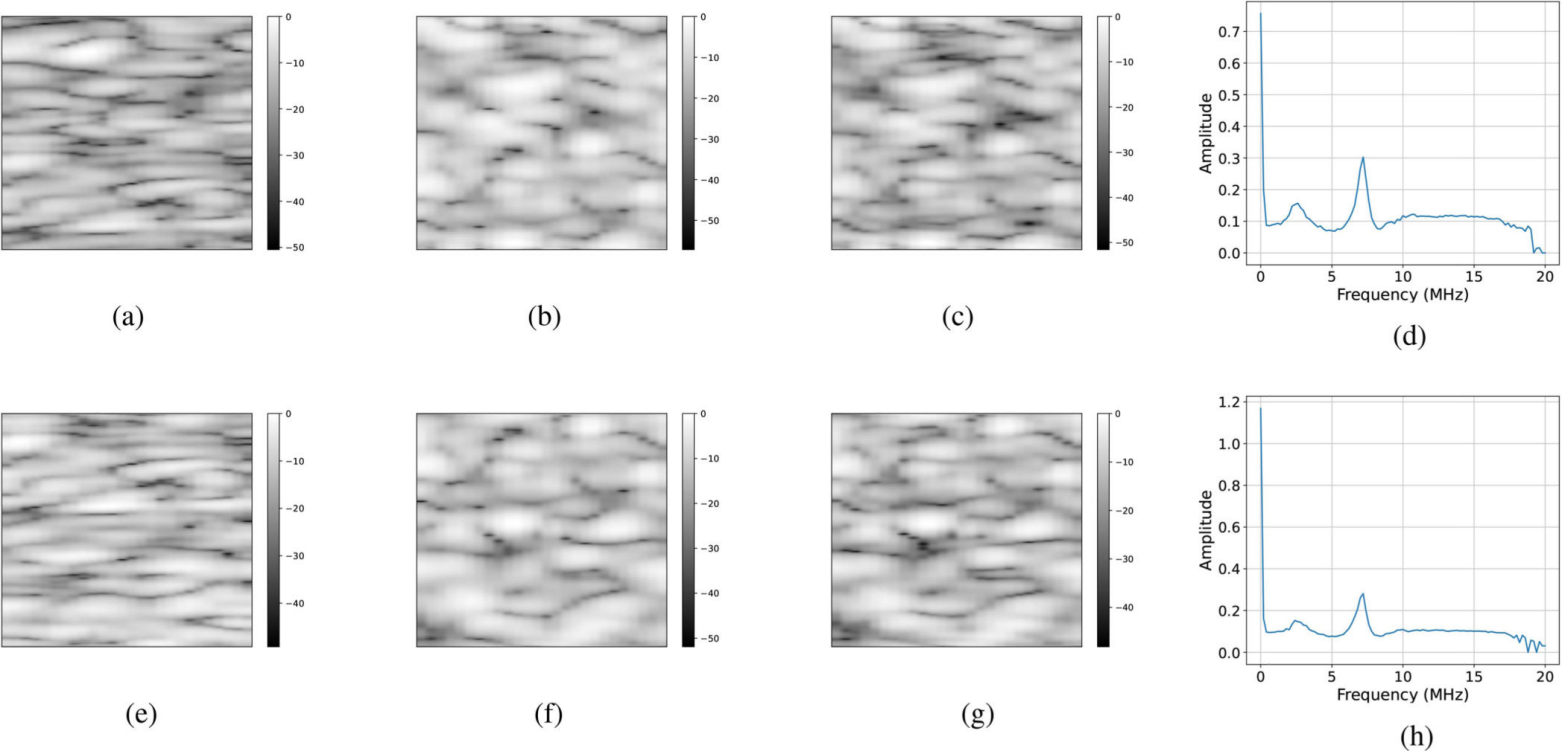
Example B-mode Ultrasound Image Patches Before and After Applying the Transfer Function: Each row presents a set of examples for $X_{victim}$, $X_{perpetrator\;}$, $X_{perpetrator\;\to \;victim\;}$ and Γ. The first column shows $X_{victim}$, second column shows $X_{perpetrator\;}$, third column shows $X_{perpetrator\;\to \;victim\;}$ and the fourth column shows Γ.

We plotted two sets of example B-mode ultrasound image patches from $X_{victim}$, $X_{perpetrator\;}$ and $X_{perpetrator\;\to \;victim\;\;}$, along with the respective transfer function before and after applying the transfer function. Example B-mode image patches can be found in Fig. 5. Quantitatively, it can be observed that the speckle pattern in $X_{perpetrator\;}$ gets closer to $X_{victim}$ thanks to the transfer function. In these results, to obtain examples from $X_{victim}$, the SonixOne system with the L9–4 transducer was utilized. For obtaining examples from $X_{perpetrator\;}$, the Verasonics system with the L9–4 transducer was utilized. These image patches were from the centrallateralposition,andinterms of depth, they were from the focal area.

## IV. DISCUSSION

In this study, the proposed approach utilized a recently emerged transfer function within an iterative learning schema. The transfer function aids in decreasing noise in the pseudo-labeling process, while the iterative schema further refines the labels. The study’s key observation highlights the ease with which the functionality of DL-based QUS methods can be stolen, raising valid concerns regarding model security. The ease with which a competitor company can transfer this functionality to its own machines poses a risk, potentially causing delays in deploying DL-based QUS methods in clinical settings. Therefore, as an outcome of the study, we emphasize the need for a deeper understanding of model security and the development of secure deployment methods tailored for ultrasound imaging contexts. Developing techniques to safeguard machine learning models emerges as a critical challenge in the adoption of wider clinical applications of machine learning-based algorithms. As of today, if one can estimate a transfer function between machines and assuming one can access the input-output interface of a victim ultrasound machine, there is no known defense against such model extraction attacks [36].

The study primarily aligns with the black-box UDA framework, wherein we lacked access to the internal workings of the victim model and solely utilized its input-output interface. Leveraging this interface, we obtained pseudo-labels from the unlabeled data of the perpetrator machine. Using these pseudo-labels, we were able to train the DL model on the perpetrator machine without the need for annotation and labeling of the data by an outside expert. Our primary aim was to demonstrate the ease of replicating the functionality of the victim machine. While there might be technical differences such as model architecture between the victim and perpetrator machines, our focus remained on the functionality, considering it as the pivotal aspect of our investigation.

In Table III, we examined the impact of integrating the transfer function within the iterative schema. The findings highlighted a significant positive impact of the transfer function on the performance. In the absence of the transfer function, the iterative schema yielded accuracy scores ranging between 85–90 percent. However, upon incorporating the transfer function, the performance consistently surged to the 98 percent range across all denoising cycle numbers. Its impact on the AUC score also demonstrated a positive effect, elevating the AUC score to 0.998. This table illustrated the substantial contribution brought by the transfer function to the iterative schema. While the study focused on the utilization of the IterLNL schema, the transfer function holds the potential to similarly benefit all other black-box UDA methods.

In Table IV, we explored the effects of the prior about the label distribution. We observed that the proposed method demonstrated robust performance across various scenarios, maintaining an accuracy score consistently above 97 percent and an AUC score of 0.997. As a future direction, it is possible to explore various problem scenarios, including imbalanced classification, to increase the impact of the proposed approach. Moreover, the proposed approach can be scaled to a multi-class problem setting, as in certain clinical tasks, there may be multiple types of tissue states. As long as we have access to the input-output interface and obtain pseudo-labels in a multi-class setting, we expect the proposed approach to scale well.

In Table V, we investigated the effects of the prior about the noise rate ϵ. The noise rate plays a crucial role in the iterative schema, influencing the selection of anchors at each training epoch based on this parameter. Given the prior work on transfer function approach, the noise rate was estimated at 20 percent so that 80 percent of the data was utilized as anchors during the training. In Table V, we investigated scenarios involving inaccurate or approximate prior knowledge of the noise rate such as 10 and 30 percent during the acquisition of anchors. We observed that the proposed method demonstrated robust performance across various scenarios, maintaining an accuracy score consistently above 97 percent and an AUC score of 0.993.

In Fig. 4, we investigated the number of ultrasound frames needed from the perpetrator machine to achieve a successful attack. We used 25, 50, 125, 250, 500 and 1000 frames in the figure. Classification accuracies on the perpetrator were calculated by first performing patch extraction, obtaining pseudo-labels, refining the labels, and training a machine learning model for the perpetrator machine. Then, using a test set, we obtained classification accuracies. We observed that by just utilizing 125 ultrasound frames from the perpetrator machine, the proposed approach achieved a 94.48 percent classification accuracy. Developing a more data-efficient attack could be another direction for research.

In Table VI, we investigated the effect of using different transducers between the victim and the perpetrator machines. This scenario is relevant, as different machines could use their own customized transducers. In such a scenario, the proposed algorithm needs to be robust against these variations. We achieved a 97 percent classification accuracy and a 0.9948 AUC score on the perpetrator machine. However, the performance degraded as the number of denoising cycles increased, similar to the case when the transfer function was neglected in the approach. This seems counterintuitive as one would expect performance to increase with more denoising cycles. However, pseudo-labels become noisier rather than improving, and degradation occurs when increasing the denoising cycle number. One could likely improve the performance by tuning the hyper-parameters such as epoch number, learning rate, or noise rate. Such work is left as a future investigation to explore the relationships between hyper-parameters and the accuracy of the algorithm.

Although the proposed approach does not require any expert annotation, it assumes the availability of unlabeled data from the perpetrator machine. Depending on the clinical task, acquiring the unlabeled data could be as costly as acquiring expert annotation, as it requires finding the right type of patients, addressing privacy issues, and conducting lab experiments. Furthermore, as a follow-up research direction, combining data with labels acquired from the victim machine with a small subset of expert-annotated data from the perpetrator machine could be interesting and could lead to higher accuracy and more efficient attacks.

The proposed method achieved a remarkable 98 percent classification accuracy and 0.998 AUC score on the perpetrator machine by solely using the unlabeled data from the perpetrator machine and the input-output interface of the victim model. It is important to note that our assumptions included the ease with which the perpetrator machine could utilize the input-output interface, converting its data into the appropriate format and generating pseudo-labels. Furthermore, under the hood, we assumed the perpetrator has complete knowledge about the clinical task that the victim model is trained for. Therefore, the perpetrator acquires meaningful unlabeled data for the victim model. For instance, if the victim model characterizes tissue for certain organs and for certain patient distribution, we assumed the perpetrator has the complete knowledge of it. Implementing security enhancements might involve measures like hiding or restricting access to the input-output interface and developing encrypted data formats. These issues need to be revisited from a DL perspective and further researched. Nevertheless, there remains a risk of hacking, especially in a development environment if a competitor gains ownership of the victim machine. An intriguing approach to improving model security could involve integrating security measures directly into the model’s behavior, with the aim of deceiving bad actors by presenting misleading functionalities. However, in practice, implementing this type of security feature could lead to a decrease in performance for legitimate users. This remains an interesting direction for future research.

In this work, it was assumed that competing companies have the capability and resources to access the raw data sufficient to develop a transfer function, e.g., a research interface access. In scenarios where competing companies lack the necessary capabilities and resources, it would be interesting to implement the idea of combining the transfer function and iterative refinement in the image data domain, where it does not require access to raw data, such as MimickNet [24]. Performance-wise, implementing this approach in the image data domain is expected to have lower accuracies, but iterative refinement can be improved further to compensate for that. This would be an important avenue for further research. In essence, this study underscores the critical need for an enhanced understanding of model security within the domain of DL-based ultrasound imaging. Hence, we identify model security as a critical future direction encompassing two key aspects: first, the development of new strategies to transfer DL-based functionalities to the perpetrator machine; and second, the development of security measures to defend against these potential attacks. Furthermore, the developed approach can also be a useful tool for regulatory bodies, especially when procedures require openness. They can use the proposed approach to obtain open regulatory models that can be used during the approval process. The dataset for this work can be accessed using the link: https://uofi.box.com/s/d9ecw002ree6gj9tlplz7t0i2f1ojbk7, and the code for this study is available at the repository: https://github.com/usoylu2/theartsteal.

## V. CONCLUSION

We demonstrated the ability to adeptly acquire the functionality of a DL QUS model from a machine. This capability can be utilized by regulatory bodies for DL model regulation, while simultaneously raising valid concerns regarding model security. The ease of transferring functionality to competitors poses a significant risk to future DL-based development in diagnostic ultrasound. The study emphasizes the need for a deeper understanding of model security and the development of secure deployment methods tailored for DL-based ultrasound imaging.

## Figures and Tables

**TABLE I:** Victim Model Architecture: DenseNet-201

stage	output	kernels
convl	102 × 15	7 × 7, 64, stride 2
dense1	51 × 8	3 × 3 max pool, stride 2
		$\left[\begin{array}{l} 1\times 1\\ 3\times 3 \end{array}\right]\times 6$
tr1	25 × 4	1 × 1, 256 and 2 × 2 avgpool, stride 2
dense2	25 × 4	$\left[\begin{array}{l} 1\times 1\\ 3\times 3 \end{array}\right]\times 12$
tr2	12 × 2	1 × 1, 512 and 2 × 2 avgpool, stride 2
dense3	12 × 2	$\left[\begin{array}{l} 1\times 1\\ 3\times 3 \end{array}\right]\times 48$
tr3	6 × 1	1 × 1, 1024 and 2 × 2 avgpool, stride 2
dense4	6 × 1	$\left[\begin{array}{l} 1\times 1\\ 3\times 3 \end{array}\right]\times 32$
	1 × 1	global average pool 2-d fc, softmax

**TABLE II:** Perpetrator Model Architecture: ResNet-34

stage	output	kernels
conv1	100 × 13	7 × 7, 64, stride 2
conv2	50 × 7	3 × 3 max pool, stride 2
		$\left[\begin{array}{l} 3\times 3,64\\ 3\times 3,64 \end{array}\right]\times 3$
conv3	25 × 4	$\left[\begin{array}{l} 3\times 3,128\\ 3\times 3,128 \end{array}\right]\times 4$
conv4	13 × 2	$\left[\begin{array}{l} 3\times 3,256\\ 3\times 3,256 \end{array}\right]\times 6$
conv5	7 × 1	$\left[\begin{array}{l} 3\times 3,512\\ 3\times 3,512 \end{array}\right]\times 3$
	1 × 1	global average pool 2-d fc, softmax

**TABLE III:** Ablation Study for the Transfer Function

No	Experiment	Accuracy (Cycle=2)	AUC (Cycle=2)	Accuracy (Cycle=5)	AUC (Cycle=5)	Accuracy (Cycle=10)	AUC (Cycle=10)
1	Iterative Learning with Transfer Function	**97.83±0.28**	**0.9976±5e-4**	**97.78±0.25**	**0.9975±5e-4**	**97.69±0.36**	**0.9974±7e-4**
2	Iterative Learning without Transfer Function	89.98±3.88	0.9618±3e-2	89.22±7.39	0.9401±6e-2	85.51±1.60	0.9020±1e-1

**TABLE IV:** Robustness Experiment With Label Distribution Prior

No	Experiment	Accuracy (Cycle=2)	AUC (Cycle=2)	Accuracy (Cycle=5)	AUC (Cycle=5)	Accuracy (Cycle=10)	AUC (Cycle=10)
1	40^*th*^ percentile	97.35±1.44	0.9975±7e-4	97.71 ±0.33	0.9974±6e-4	97.70±0.51	0.9975±7e-4
2	50^*th*^ percentile	**97.83±0.28**	**0.9976±5e-4**	**97.78±0.25**	**0.9975±5e-4**	97.69±0.36	0.9974±7e-4
3	60^*th*^ percentile	97.55±0.47	0.9974±5e-4	97.48±0.46	0.9972±7-e4	**97.82±0.38**	**0.9976±7e-4**

**TABLE V:** Robustness Experiment With Noise Rate ϵ Prior

No	Experiment	Accuracy (Cycle=2)	AUC (Cycle=2)	Accuracy (Cycle=5)	AUC (Cycle=5)	Accuracy (Cycle=10)	AUC (Cycle=10)
1	10 percent	**98.25±0.29**	**0.9983±5e-4**	**98.13±0.28**	**0.9982±4e-4**	**98.25±0.28**	**0.9983±5e-4**
2	20 percent	97.83±0.28	0.9976±5e-4	97.78±0.25	0.9975±5e-4	97.69±0.36	0.9974±7e-4
3	30 percent	97.60±0.82	0.9969±2e-3	97.02±1.57	0.9954±4e-3	96.45±2.46	0.9932±1e-2

**TABLE VI:** Transducer Mismatch Between the Victim and the Perpetrator Machine

Denoising Cycle	Accuracy	AUC
2	**96.87±1.39**	**0.9948±4e-3**
5	93.55±5.00	0.9718±3e-2
10	88.53±5.81	0.9391±5e-2
